# Associations between NLRC4 Gene Polymorphisms and Autoimmune Thyroid Disease

**DOI:** 10.1155/2020/1378427

**Published:** 2020-08-04

**Authors:** Xuerong Liu, Xiaogang Bai, Jing Zhao, Chaoqun Gao, Peng Du, Jin-an Zhang, Sheli Li

**Affiliations:** ^1^Department of Endocrinology, Yanan University Affiliated Hospital, Shaanxi 716000, China; ^2^Department of Endocrinology, Shanghai University of Medicine & Health Sciences Affiliated Zhoupu Hospital, Shanghai 201508, China

## Abstract

**Background:**

Many studies have shown that NLRC4 inflammasome polymorphisms are associated with a variety of autoimmune diseases, but the associations between NLRC4 polymorphisms and autoimmune thyroid diseases (AITDs) are unclear. Our research was aimed at identifying the correlations between NLRC4 polymorphisms and AITDs.

**Methods:**

Hi-SNP high-throughput genotyping technology was used for detecting four single-nucleotide polymorphisms (SNPs) of NLRC4 in 1005 AITDs patients (including 629 Graves' disease and 376 Hashimoto's thyroiditis) and 781 healthy controls.

**Results:**

Compared with healthy controls, the allele frequencies and genotype distribution of rs385076 were statistically related to AITDs (*P* = 0.016 and *P* = 0.048, respectively) and Hashimoto's thyroiditis (*P* = 0.022 and *P* = 0.046, respectively). Before adjusting for age and gender, rs385076 and AITDs had a significant association in three models of allele model, dominant model, and homozygous model. After adjusting for age and gender, in the above three models, there is still a clear relationship between them. Before adjusting for age and gender, there were prominent discrepancy between rs385076 and Hashimoto's thyroiditis in the allele model (OR = 0.81, 95% CI 0.67-0.97; *P* = 0.021) and the dominant model (OR = 0.73, 95% CI 0.57-0.94; *P* = 0.014), after adjusting for age and gender, rs385076 and Hashimoto's thyroiditis were significantly related to allele model, dominant model, and homozygous model. However, rs455060, rs212704, and rs675712 were not related to AITDs in our study.

**Conclusion:**

NLRC4 rs385076 was found to have a significant association with Hashimoto's thyroiditis for the first time. It laid a foundation for the disclosure of the pathogenesis of AITDs, and provided a possible treatment prospect for HT.

## 1. Introduction

Autoimmune thyroid diseases (AITDs) are not a disease but a group of diseases; the main damage is not only the thyroid gland but also the other parts of the body. AITDs mainly include two types of Graves' disease (GD) and Hashimoto's thyroiditis (HT) [1]. GD is characterized by an abnormal increase in thyroid-stimulating hormone (TSH) receptor antibody (TRAb), which competitively binds to the TSH receptor (TSHR) on thyroid follicular cells with TSH, resulting in increased synthesis and secretion of thyroid hormones [1, 2]. The main feature of HT is the abnormal increase in thyroid peroxidase antibody (TPOAb) and thyroglobulin antibody (TgAb). TPOAb can trigger complement-dependent cytotoxicity, damage thyroid cells, and lead to insufficient thyroid hormone secretion [2]. GD and HT have the exact same pathological characteristics that are lymphocyte infiltration in thyroid tissue and antibodies production, so they have similar pathogenesis [3]. At present, the pathogenesis of AITDs is unclear, but most believe that environmental factors and hereditary factors play a crucial role in AITDs [4, 5]. Epidemiological surveys show that the prevalence of GD in China is about 0.8%, and the prevalence of HT is as high as 10% or more; the global prevalence of AITD is approximately 5%, with about 10 million GD patients and more than 130 million HT patients [6–8]. Patients with AITD increase in cardiovascular and tumor risks, increase the likelihood of adverse pregnancy such as miscarriage and premature birth, and seriously endanger public health [1, 9]. However, the current treatment methods are very limited, especially the treatment methods for the etiology are lacking.

Inflammasome is a multiprotein complex, which is activated by infection and injury and then which promotes the maturation of proinflammatory factors and participates in the innate immune response [10]. Inflammasomes are composed of three parts: (1) adaptor protein, that is, apoptosis-associated speck-like protein contains caspase activation recruitment domain (ASC); (2) caspase-1(CASP1), after activation; pro-IL-*β* and pro-IL-18 are sheared to form mature IL-1*β* and IL-18; (3) absent in melanoma 2- (AIM-2-) like receptor (ALR) or NOD-like receptor (NLR) forming stress signal receptors or framework proteins [10–12]. NLR family caspase recruitment domain- (CARD-) containing 4 (NLRC4) belongs to the NLR family and is mainly activated by Gram-negative bacteria containing a type III or type IV secretion system [13]. When infected with Salmonella and Legionella, NLRC4 inflammasomes are activated by recognizing their flagellins and T3SS proteins [14–16]. The activated NLRC4 N-terminal domain CARD directly interacts with pro-CASP1 to generate CASP1 and simultaneously produces two active subunits—P10 and P20 tetramers. The CASP-1 P20 subunit medicates the conversion of pro-IL-*β* and pro-IL-18 to IL-*β* and IL-18 [17]. Subsequently, CASP1 activates the Gasdermin-D protein, and the N-terminal structure of NLRC4 after cleavage of this protein acts on the host cytomembrane, leading to the loss of cytomembrane integrity, eventually inducing pyroptosis; inflammatory factors such as IL-*β* and IL-18 are released to the outside of cell through the membrane pores formed by Gasdermin-D, promoting the body to resist pathogens [18].

Studies have confirmed that NLRC4 gene polymorphisms may be related to the development and severity of multiple autoimmune diseases [19–21]. One study found that rs385076 plays an extremely crucial role in NLRC4 expression and differentiation [22]. This study intends to analyze the relationship between the four SNPs (rs385076, rs455060, rs212704, and rs675712) of NLRC4 and genetic susceptibility to AITD in a large sample case-control study.

## 2. Methods

### 2.1. Subjects

A total of 1786 subjects were recruited in the current study (including 629 GD patients, 376 HT patients, and 781 healthy controls). The AITD patients were recruited from the endocrinology clinic of Shanghai University of Medicine & Health Sciences Affiliated Zhoupu Hospital. Patients with other chronic diseases and severe diseases were excluded from the case group. Healthy controls were from health check-up centers of the same hospital. And those with other autoimmune diseases, chronic diseases and with family history of thyroid disease were excluded from control group. All recruiters were Chinese and signed informed consent. The diagnostic criteria for GD were clinical manifestations of hyperthyroidism, positive TRAb, and diffuse goiter. The diagnostic criteria for HT are goiter, TgAb positive, or TPOAb positive, with or without hypothyroidism.

### 2.2. Genotyping

We used venipuncture to collect 2 ml of peripheral blood from each recruit and aliquoted it into anticoagulant tubes containing ethylene diamine tetraacetic acid (EDTA). According to the manufacturer's instructions, the RelaxGene Blood DNA System (Tiangen Biotech, Beijing, China) was used to extract genomic DNA, and then, the A260/280 ratio and concentration of the extracted genomic DNA samples were detected. Using the following three criteria: (1) minor allele frequency (MAF) > 0.05; (2) HardyWeinberg equilibrium (HWE) > 0.01; and (3) logarithm of odds (LOD) > 3.0, we screened the SNPs of the NLRC4 gene from the Hapmap-CHB database. We finally screened four sites covering the entire region of the NLRC4 gene, rs385076, rs455060, rs212704, and rs675712, to capture the most common variants of this gene. Multiplex PCR and high-throughput sequencing genotyping methods were used to test these 4 SNPs [23]. Amplify the extracted DNA in a 10 *μ*l PCR thermal cycling reaction; amplification conditions are as follows: 95°C—15 minutes (1 cycle); 94°C—30 seconds, 60°C—10 minutes, 72°C—30 seconds (4 cycles); 94°C—30 seconds, 60°C—1 minutes, 72°C—30 seconds (20 cycles). Amplify the PCR products from the first round for the second PCR and continue to further amplify the PCR products from the second round [24]. The primers for amplifying the target loci are as follows: rs385076 forward 5′-AATGTTAAGGCTTTTGTACATCCC-3′ reverse 5′-AAGGTATCTGGTCTACAAGAACTC-3′; rs455060 forward 5′-TCCAGTTGAAGAACAAAGATACAG-3′ reverse 5′-GGAACATCCCCGATTACTTATTTG-3′; rs212704 forward 5′-AAAGTAAACACCACCTAACTGTTG-3′ reverse 5′TATCAGAAAGACAGGATATCCACC-3′; and rs675712 forward 5′-CTTCCAGATAGGCCAGATTCAG-3′ reverse 5′AGATTTTGCCAGTGTCAATCAAG-3′.

### 2.3. Statistical Analysis

This study used STATA 15.0 statistical software for statistical analysis. Hardy-Weinberg equilibrium (HWE) evaluation uses *χ*^2^ test. *χ*^2^ test was also used to compare the allele frequency and genotype distribution between the case group and the control group. Multiple gene association models were used to analyze the relation between SNPs of the NLRC4 gene and AITD. Multivariate logistic regression analysis was used to correct confounding factors, and the odds ratio (OR) and 95% confidence interval (95% CI) were estimated. *P* < 0.05 indicates statistical significance.

## 3. Results

### 3.1. Subjects

Our study recruited 629 patients with GD, 376 patients with HT, and 781 healthy controls. In the GD group, there were 189 males (30.05%) and 440 females (69.95%), and the average age of GD patients was 40.82 ± 14.63 years. There were 132 cases (20.99%), 166 cases (26.39%), 289 cases (45.94%), and 42 cases (6.68%) of those without goiter, with I degree, II degree, and III degree goiter, respectively, and 132 cases (20.99%) had a family history of AITD, and 99 cases (15.74%) had ophthalmopathy. In the HT group, there were 54 males (14.36%) and 322 females (85.64%), and the average age of HT recruits was 42.20 ± 14.08 years. There were 158 cases (42.02%), 92 cases (24.47%), 119 cases (31.65%), and 7 cases (1.86%) of those without goiter, with I degree, II degree, and III degree goiter, respectively, and 61 patients (16.22%) had a family history of AITD, and 3 patients (0.80%) had ophthalmopathy. There were 312 males (39.95%) and 469 females (60.05%) in the healthy control group, with an average age of 39.34 ± 10.11 years (the above results are shown in Table 1). There were no significant differences in the *P* values of the HWE test at the rs385076, rs455060, rs212704, and rs675712 between the AITD group and the control group (*P* > 0.05), indicating that the research subjects included in this study were group representative.

### 3.2. Allelic and Genotypic Analysis

Allele frequencies and genotype distribution of NLRC4 polymorphism are given in Table 2. At rs385076, the frequencies of TT, TC, and CC alleles were 34.06%, 50.45%, and 15.49% in the healthy control group, 39.20%, 48.06%, and 12.74% in the AITD group, 37.84%, 49.60%, 12.56% in the GD group, and 41.49%, 45.48%, 13.03% in the HT group, respectively. We can see from Table 2, for rs385076, that the allele frequencies and genotype distribution in the healthy control group were significantly different from those in the AITD recruits (with *P* values of 0.016 and 0.048) and HT group (with *P* values of 0.022 and 0.046). The frequency of allele T of rs385076 (62.64%) in GD patients was higher than that in controls (59.28%), although this association failed to reach significant probabilities (*P* = 0.070). At rs455060, rs212704, and rs765712 of NLRC4, compared with the healthy control group, the AITD group, GD cases, and HT patients all had *P* values greater than 0.05, regardless of allele frequency or genotype distribution, that is, there was no significant discrepancy between these case group and control group. In order to further investigate the associations between NLRC4 and AITDs, we analyzed these data in four different gene association models. In Table 3, before adjusting for confounding factors, including age and gender, the rs385076 of NLRC4 and AITD had significant differences in the allele model, dominant model, and homozygous model, and the ORs were 0.84 (95% CI 0.73-0.97, *P* = 0.014), 0.80 (95% CI 0.66-0.97, *P* = 0.026), 0.85 (95% CI 0.73-0.98, *P* = 0.024), respectively. Through multiple logistic regression analysis, after adjusting for age and gender, there were still significant differences in the three models. In all gene association analysis models, there was no significant finding between AITD and healthy controls in rs455060, rs212704, and rs765712 of NLRC4 before and after adjusting for age and gender (Table 3).

As given in Table 4, the rs385076 of NLRC4 in the GD group were not significantly different in the any models before and after adjusting for age and gender (*P* > 0.05). In Table 5, the rs385076 had significant correlations with HT only in the allele model (*P* = 0.021) and the dominant model (*P* = 0.014) before adjusting for age and gender. Through multivariate analysis, after adjusting confounding factors, the allele model (OR = 0.79, 95% CI 0.65-0.95, *P* = 0.013), the dominant model (OR = 0.72, 95% CI 0.55-0.94, *P* = 0.014), and homozygous model (OR = 0.80, 95% CI 0.65-0.98, *P* = 0.028) still have significant differences. In all comparison models, rs455060, rs212704, and rs765712 of NLRC4 are also neither related to GD nor HT (Tables 4 and 5).

### 3.3. Correlations between Genotype and Clinical Subphenotype

According to the clinical characteristics, we analyzed the correlation of clinical subtype among all diseased individuals, which were mainly divided into (1) age of onset ≤18 years versus age of onset ≥19 years; (2) GD patients with exophthalmos versus GD without exophthalmos; and (3) with hypothyroidism versus without hypothyroidism in HT group. The allele or genotype distribution of the 4 SNPs of the NLRC4 gene is not related to the above three clinical phenotypes (Tables 6, 7, and 8).

## 4. Discussion

Previous studies have shown that human and rat thyroid cells express a variety of functional Toll-like receptors (TLR) on the surface; these receptors recognize endogenous and exogenous threats and activate the innate immune system [25, 26]. Innate immune response is linked to the destruction of thyroid tissues. After the tissue cells are injured, intracellular materials are released. These substances can be recognized by antigen-presenting cells [27], then acquired immune responses are activated, which further damages thyroid cells and eventually leads to thyroid dysfunction [28–30].Under exogenous and endogenous stress conditions, inflammasome can recognize pathogen-associated molecular patterns (PAMPs) and endogenous damage-associated patterns (DAMPs) [31], recruit, and activate biologically active CASP1-1, and subsequently CASP1 can convert nonbiologically active pro-IL-1*β* and pro-IL-18 into active IL-1*β* and IL-18, immune response ensues [10, 17, 18]. NLRC4 is the core component of inflammasome, which can promote the production of CASP1-dependent proinflammatory cytokines and pyroptosis to participate in the immune response [32, 33]. Both NLRC4 and IL-18 take parts in a variety of inflammatory and autoimmune diseases [22]. Many studies have confirmed that NLRC4 are associated with multiple autoimmune diseases such as RA (rheumatoid arthritis) [19], MS (multiple sclerosis) [21, 34], and psoriasis [35]. Reports have confirmed that in patients with RA, NLRC4 expression was enhanced in monocytes after treatment with lipopolysaccharide and ATP [19]; G > C mutation at rs479333 of NLRC4 plays a beneficial role in the progression of MS and the response after interferon treatment [21]; through Genome-Wide Association Studies (GWAS) and various omics studies, Zeller et al. [22] demonstrated that rs385076 of NLRC4 has a fatal role in regulating the expression and differentiation of NLRC4 by regulating its binding to the transcription factor PU.1 and is related to the activation of IL-18.

This study explored the correlation between the four SNPs (rs385076, rs455060, rs212704, and rs765712) of NLRC4 and AITD. We found that the frequency of the allele T of rs385076 in HT patients was significantly higher than that of the control group (64.23% vs. 59.28%). Before and after adjusting for age and gender, the three comparison models of allele model, dominant model, and homozygous model have significant correlation with HT and are a protective factor. The above results indicate that the NLRC4 gene is related to HT and provides new genetic evidence for HT. NLRC4 is involved in innate immunity and acquired immune processes. This gene polymorphism will lead to immune dysfunction, resulting in further damage to the thyroid due to infiltration of immune cells. Although the function of NLRC4 has not yet been fully elucidated, this gene may be involved in the pathogenesis of HT. The possible pathogenic role of NLRC4 in HT is to induce lymphocytes infiltration in thyroid by mediating pyroptosis and release of inflammatory cytokines after activation of inflammasome, which promotes the expression of inflammatory components in thyroid and prolong the immune response, eventually damages the thyrocytes. Interestingly, Guo and his colleagues [11] confirmed that NLRC4 and its downstream cytokines (including IL-1*β* and IL-18) are increased in patients with HT, and immunohistochemistry found that those proteins are mainly overexpressed in the vicinity of thyroid follicular cells infiltrated by lymphocytes, and in contrast, these proteins are under expressed near follicles without lymphocyte infiltration. The above findings support our findings directly, but the mechanism still needs to be further explored. We found no correlation between the 4 SNPs of NLRC4 and GD, indicating that these 4 SNPs are not related to the incidence of GD. Although GD and HT are similar autoimmune diseases that occur in the thyroid, their pathogenesis and etiology are not exactly the same [36], which explains the different association between NLRC4 polymorphism and GD and HT.

After subgroup analysis based on clinical phenotype, we found no correlation between cases with clinical phenotype and cases without clinical phenotype. The possible reason for not finding the correlation is stratification leading to genetic heterogeneity or too few samples. It is recommended to use a large sample case-control study to evaluate the relationship between NLRC4 gene and GD more accurately.

In summary, for the first time, we found that the four SNPs of the NLRC4 gene is associated with HT, but not associated with GD. Although both GD and HT are caused by lymphocytes infiltrating the thyroid gland, their pathogenesis is not exactly the same [5, 36]. A larger sample of case-control studies is needed to further determine these observations.

## Figures and Tables

**Table 1:** Clinical and demographics features of the subjects.

Items	AITD	GD	HT	Controls
Number	1005	629	376	781
Gender				
Male	243 (24.18%)	189 (30.05%)	54 (14.36%)	312 (39.95%)
Female	762 (75.82%)	440 (69.95%)	322 (85.64%)	469 (60.05%)
Age (years)	41.34 ± 14.44	40.82 ± 14.63	42.20 ± 14.08	39.34 ± 10.11
Goiter				
No goiter	290 (28.86%)	132 (20.99%)	158 (42.02%)	—
Degree I	258 (25.67%)	166 (26.39%)	92 (24.47%)	—
Degree II	408 (40.59%)	289 (45.94%)	119 (31.65%)	—
Degree III	49 (4.88%)	42 (6.68%)	7 (1.86%)	—
Family history		—		
(+)	193 (19.20%)	132 (20.99%)	61 (16.22%)	—
(-)	812 (80.80%)	497 (79.01%)	315 (83.78%)	—
Ophthalmopathy		—		
(+)	102 (10.15%)	99 (15.74%)	3 (0.80%)	—
(-)	903 (89.85%)	530 (84.26%)	373 (99.20%)	—

GD: Graves' disease; HT: Hashimoto's thyroiditis.

**Table 2:** Allele frequencies and genotype distribution of NLRC4 polymorphisms in AITD patients and controls.

Gene/SNP	Controls	AITD	*P* value(OR, 95% CI)	GD	*P* value(OR, 95% CI)	HT	*P* value(OR, 95% CI)
NLRC4	*n* (%)	*n* (%)	AITD vs. control	*n* (%)	GD vs. control	*n* (%)	HT vs. control
rs385076							
T	926(59.28)	1271(63.23)	0.016(0.847,0.739-0.970)	788(62.64)	0.070(0.868,0.746-1.011)	483(64.23)	0.022(0.811,0.677-0.971)
C	636(40.72)	739(36.77)		470(37.36)		269(35.77)	
TT	266(34.06)	394(39.20)	0.048-	238(37.84)	0.169-	156(41.49)	0.046-
TC	394(50.45)	483(48.06)		312(49.60)		171(45.48)	
CC	121(15.49)	128(12.74)		79(12.56)		49(13.03)	
rs455060							
A	715(45.77)	872(43.38)	0.154(1.102,0.964-1.258)	547(43.48)	0.224(1.097,0.945-1.274)	325(43.22)	0.247(1.109,0.931-1.322)
G	847(54.23)	1138(56.62)		711(56.52)		427(56.78)	
AA	160(20.49)	181(18.01)	0.335-	109(17.33)	0.322-	72(19.15)	0.421-
AG	395(50.58)	510(50.75)		329(52.31)		181(48.14)	
GG	226(28.93)	314(31.24)		191(30.36)		123(32.71)	
rs212704							
T	718(45.97)	871(43.33)	0.116(1.112,0.974-1.271)	546(43.40)	0.173(1.109,0.955-1.288)	325(43.22)	0.213(1.118,0.938-1.332)
C	844(54.03)	1139(56.67)		712(56.60)		427(56.78)	
TT	164(21.00)	180(17.91)	0.23-	111(17.65)	0.28-	69(18.35)	0.455-
TC	390(49.93)	511(50.85)		324(51.51)		187(49.73)	
CC	227(29.07)	314(31.24)		194(30.84)		120(31.92)	
rs675712							
A	923(59.09)	1184(58.91)	0.911(1.008,0.881-1.153)	737(58.59)	0.786(1.021,0.878-1.187)	447(59.44)	0.872(0.986,0.826-1.177)
G	639(40.91)	826(41.09)		521(41.41)		305(40.56)	
AA	276(35.34)	361(35.92)	0.783-	228(36.25)	0.497-	133(35.37)	0.957-
AG	371(47.50)	462(45.97)		281(44.67)		181(48.14)	
GG	134(17.16)	182(18.11)		120(19.08)		62(16.49)	

AITD: autoimmune thyroid disease; GD: Graves' disease; HT: Hashimoto's thyroiditis.

**Table 3:** Odds ratios (ORs) of the associations of four polymorphisms in the NLRC4 gene with AITD before and after adjusting for confounders (age and gender).

Comparison models	Unadjusted estimates		Adjusted estimates^∗^	
	OR (95% CI)	*P* values	OR (95% CI)	*P* values
rs385076				
Allele model	0.84 (0.73-0.97)	0.014	0.84 (0.73-0.97)	0.014
Dominant model	0.80 (0.66-0.97)	0.026	0.80 (0.66-0.97)	0.027
Recessive model	0.80 (0.61-1.04)	0.096	0.79 (0.60-1.04)	0.091
Homozygous model	0.85 (0.73-0.98)	0.024	0.84 (0.72-0.98)	0.023
rs455060				
Allele model	0.91 (0.79-1.04)	0.148	0.91 (0.79-1.04)	0.169
Dominant model	0.90 (0.73-1.10)	0.293	0.91 (0.74-1.11)	0.348
Recessive model	0.85 (0.67-1.08)	0.187	0.85 (0.67-1.08)	0.183
Homozygous model	0.90 (0.79-1.03)	0.140	0.90 (0.79-1.04)	0.154
rs212704				
Allele model	0.90 (0.78-1.03)	0.112	0.90 (0.79-1.04)	0.149
Dominant model	0.90 (0.74-1.11)	0.320	0.92 (0.74-1.13)	0.403
Recessive model	0.82 (0.65-1.04)	0.101	0.83 (0.65-1.05)	0.117
Homozygous model	0.89 (0.78-1.02)	0.095	0.90 (0.78-1.03)	0.122
rs675712				
Allele model	1.01 (0.88-1.15)	0.913	1.02 (0.89-1.17)	0.760
Dominant model	0.97 (0.80-1.19)	0.799	1.00 (0.83-1.23)	0.965
Recessive model	1.07 (0.84-1.37)	0.601	1.07 (0.83-1.37)	0.610
Homozygous model	1.02 (0.89-1.17)	0.786	1.03 (0.90-1.18)	0.688

AITD: autoimmune thyroid disease; OR: odds ratio; 95% CI: 95% confidence interval; ^∗^age and gender were adjusted in the multivariate logistic regression analyses.

**Table 4:** Odds ratios (ORs) of the associations of four polymorphisms in the NLRC4 gene with GD before and after adjusting for confounders (age and gender).

Comparison models	Unadjusted estimates		Adjusted estimates^∗^	
	OR (95% CI)	*P* values	OR (95% CI)	*P* values
rs385076				
Allele model	0.86 (0.74-1.01)	0.063	0.86 (0.73-1.01)	0.061
Dominant model	0.85 (0.68-1.06)	0.141	0.84 (0.68-1.05)	0.130
Recessive model	0.87 (0.58-1.06)	0.117	0.79 (0.58-1.07)	0.125
Homozygous model	0.85 (0.72-1.01)	0.064	0.85 (0.72-1.01)	0.063
rs455060				
Allele model	0.91 (0.78-1.06)	0.215	0.91 (0.78-1.06)	0.235
Dominant model	0.93 (0.74-1.18)	0.559	0.94 (0.75-1.18)	0.598
Recessive model	0.81 (0.62-1.07)	0.134	0.82 (0.62-1.07)	0.142
Homozygous model	0.90 (0.77-1.05)	0.173	0.90 (0.77-1.05)	0.185
rs212704				
Allele model	0.90 (0.77-1.05)	0.169	0.91 (0.78-1.05)	0.203
Dominant model	0.92 (0.73-1.16)	0.469	0.93 (0.74-1.17)	0.525
Recessive model	0.81 (0.62-1.05)	0.115	0.81 (0.62-1.07)	0.134
Homozygous model	0.89 (0.76-1.04)	0.138	0.89 (0.77-1.04)	0.158
rs675712				
Allele model	1.02 (0.88-1.18)	0.791	1.03 (0.89-1.19)	0.704
Dominant model	0.96 (0.77-1.20)	0.723	0.98 (0.79-1.22)	0.864
Recessive model	1.14 (0.87-1.49)	0.351	1.14 (0.86-1.50)	0.360
Homozygous model	1.04 (0.90-1.21)	0.601	1.05 (0.90-1.22)	0.540

GD: Graves' disease; OR: odds ratio; 95% CI: 95% confidence interval; ^∗^age and gender were adjusted in the multivariate logistic regression analyses.

**Table 5:** Odds ratios (ORs) of the associations of four polymorphisms in the NLRC4 gene with HT before and after adjusting for confounders (age and gender).

Comparison models	Unadjusted estimates		Adjusted estimates^∗^	
	OR (95% CI)	*P* values	OR (95% CI)	*P* values
rs385076				
Allele model	0.81 (0.67-0.97)	0.021	0.79 (0.65-0.95)	0.013
Dominant model	0.73 (0.57-0.94)	0.014	0.72 (0.55-0.94)	0.014
Recessive model	0.82 (0.57-1.17)	0.269	0.75 (0.52-1.09)	0.138
Homozygous model	0.83 (0.69-1.01)	0.060	0.80 (0.65-0.98)	0.028
rs455060				
Allele model	0.90 (0.76-1.07)	0.246	0.88 (0.73-1.05)	0.164
Dominant model	0.84 (0.64-1.09)	0.190	0.83 (0.63-1.10)	0.194
Recessive model	0.92 (0.67-1.25)	0.595	0.85 (0.62-1.18)	0.341
Homozygous model	0.91 (0.76-1.09)	0.293	0.88 (0.73-1.06)	0.180
rs212704				
Allele model	0.89 (0.75-1.07)	0.212	0.88 (0.73-1.06)	0.168
Dominant model	0.87 (0.67-1.14)	0.322	0.88 (0.66-1.16)	0.356
Recessive model	0.85 (0.62-1.16)	0.293	0.80 (0.58-1.11)	0.175
Homozygous model	0.89 (0.75-1.07)	0.211	0.87 (0.73-1.05)	0.153
rs675712				
Allele model	0.99 (0.83-1.18)	0.873	1.00 (1.84-1.21)	0.952
Dominant model	1.00 (0.77-1.29)	0.991	1.06 (0.81-1.38)	0.694
Recessive model	0.95 (0.69-1.33)	0.777	0.93 (0.60-1.32)	0.698
Homozygous model	0.98 (0.82-1.18)	0.827	0.98 (0.81-1.19)	0.865

HT: Hashimoto's thyroiditis; OR: odds ratio; 95% CI: 95% confidence interval; ^∗^age and gender were adjusted in the multivariate logistic regression analyses.

**Table 6:** Allele frequencies and genotype distribution of NLRC4 polymorphisms with onset age in AITD patients.

SNPs	≥19 years (%)	≤18 years (%)	*P* value	OR (95% CI)
rs385076				
T	1233 (63.36)	38 (59.38)	0.515	1.18 (0.71-1.97)
C	713 (36.64)	26 (40.62)		
TT	384 (39.47)	10 (31.25)	0.608	—
TC	465 (47.79)	18 (56.25)		
CC	124 (12.74)	4 (12.50)		
rs455060				
A	843 (43.32)	29 (45.31)	0.752	0.92 (0.56-1.52)
G	1103 (56.68)	35 (54.69)		
AA	176 (18.09)	5 (15.63)	0.606	—
AG	491 (50.46)	19 (59.37)		
GG	306 (31.45)	8 (25.00)		
rs212704				
T	840 (43.17)	31 (48.44)	0.402	0.81 (0.49-1.33)
C	1106 (56.83)	33 (51.56)		
TT	173 (17.78)	7 (21.88)	0.692	—
TC	494 (50.77)	17 (53.12)		
CC	306 (31.45)	8 (25.00)		
rs675712				
A	1151 (59.15)	33 (51.56)	0.225	1.36 (0.83-2.24)
G	795 (40.85)	31 (48.44)		
AA	351 (36.07)	10 (31.25)	0.327	—
AG	449 (46.15)	13 (40.63)		
GG	173 (17.78)	9 (28.12)		

AITD: autoimmune thyroid disease; OR: odds ratio; 95% CI: 95% confidence interval.

**Table 7:** Allele frequencies and genotype distribution of NLRC4 polymorphisms with ophthalmopathy in GD patients.

SNPs	Without (%)	With (%)	*P* value	OR (95% CI)
rs385076				
T	660 (62.26)	128 (64.65)	0.525	0.90 (0.657-1.239)
C	400 (37.74)	70 (35.35)		
TT	197 (37.17)	41 (41.41)	0.723	—
TC	266 (50.19)	46 (46.47)		
CC	67 (12.64)	12 (12.12)		
rs455060				
A	457 (43.11)	90 (45.45)	0.542	0.91 (0.67-1.23)
G	603 (56.89)	108 (54.55)		
AA	90 (16.98)	19 (19.19)	0.817	—
AG	277 (52.26)	52 (52.53)		
GG	163 (30.76)	28 (28.28)		
rs212704				
T	458 (43.21)	88 (44.44)	0.747	0.95 (0.70-1.29)
C	602 (56.79)	110 (55.56)		
TT	93 (17.55)	18 (18.18)	0.935	—
TC	272 (51.32)	52 (52.53)		
CC	165 (31.13)	29 (29.29)		
rs675712				
A	631 (59.53)	106 (53.54)	0.116	1.28 (0.94-1.73)
G	429 (40.47)	92 (46.46)		
AA	197 (37.17)	31 (31.31)	0.296	—
AG	237 (44.72)	44 (44.44)		
GG	96 (18.11)	24 (24.24)		

GD: Graves' disease; OR: odds ratio; 95% CI: 95% confidence interval.

**Table 8:** Allele frequencies and genotype distribution of NLRC4 polymorphisms with hypothyroidism in HT patients.

SNPs	Without (%)	With (%)	*P* value	OR (95% CI)
rs385076				
T	278 (62.90)	205 (66.13)	0.363	0.87 (0.64-1.18)
C	164 (37.10)	105 (33.87)		
TT	89 (40.27)	67 (43.22)	0.589	—
TC	100 (45.25)	71 (45.81)		
CC	32 (14.48)	17 (10.97)		
rs455060				
A	187 (42.31)	138 (44.52)	0.547	0.91 (0.68-1.23)
G	255 (57.69)	172 (55.48)		
AA	41 (18.55)	31 (20.00)	0.823	—
AG	105 (47.51)	76 (49.03)		
GG	75 (33.94)	48 (30.97)		
rs212704				
T	185 (41.86)	140 (45.16)	0.368	0.874 (0.65-1.17)
C	257 (58.14)	170 (54.84)		
TT	39 (17.65)	30 (19.35)	0.601	**—**
TC	107 (48.42)	80 (51.61)		
CC	75 (33.94)	45 (29.03)		
rs675712				
A	266 (60.18)	181 (58.39)	0.622	1.08 (0.80-1.45)
G	176 (39.82)	129 (41.61)		
AA	81 (36.65)	52 (33.55)	0.823	**—**
AG	104 (47.06)	77 (49.68)		
GG	36 (16.29)	26 (16.77)		

HT: Hashimoto's thyroiditis; OR: odds ratio; 95% CI: 95% confidence interval.

## Data Availability

The data analyzed during this study have been provided in the manuscript, and any further information can be made available upon request to the corresponding author.
